# The complete mitochondrial genome of *Laudakia wui* (Iguania; Agamidae)

**DOI:** 10.1080/23802359.2019.1627939

**Published:** 2019-07-11

**Authors:** Hai-Qun Jin, Yong Zhang, Li-Fang Peng, Shuang-Quan Duan, Song Huang

**Affiliations:** a Institute of Biodiversity Science and Geobiology, College of Science, Tibet University, Lhasa, China;; b College of Life and Environment Sciences, Huangshan University, Huangshan, China

**Keywords:** Mitogenome, *Laudakia wui*, phylogeny

## Abstract

In this study, the complete mitochondrial genome of *Laudakia wui*, has been firstly determined by shotgun sequencing. The overall length of mitogenome is 16,455 bp and contains 13 protein-coding genes, 22 transfer RNA genes, 2 ribosomal RNA genes, and 2 control regions. Majority of the genes (13 protein-coding genes, 2 rRNA and 14 tRNA) were distributed on the H-strand, in addition to the two *ND6* genes and eight tRNA genes, which were encoded on the L-strand. The phylogenetic tree was built by *L*. *wui* and 13 other related species. The DNA data presented here will be useful to study the evolutionary relationships and genetic diversity of *L*. *wui*.

*Laudakia wui* is an endemic species in the Tibet Autonomous Region of China (Zhao et al. 1999). It belongs to the genus Laudakia in the family Agamidae and has been identified as the Agama *himalayana scra* (Hu et al. 1987). During 1995–1996, Zhao Ermi observed and compared the rock lizard specimens and then found all the specimens from Bomi country, Xizang, being a new form, named as the *Laudakia wui* sp. nov. (Zhao 1998). The survival status of the *L. wui* on the Red List of China’s Vertebrates is near-threatened (Jiang et al. 2016). We predicted and described its full sequence of mitochondrial DNA to help us obtain its basic genetic information.

We attained the specimen of *L*. *wui* from Bomi County, Tibet Autonomous Region, China (Voucher numbers: LZ024). The tissue of the specimen was preserved and deposited in the refrigerator at −80 °C of the Museum of Huangshan university. The complete and circular mitochondria (Genbank accession number: MK411597) of the *L. wui* was sequenced to be 16,455 bp, which consisted of typical vertebrate 13 protein-coding genes (PCGs), 22 transfer RNA (tRNA) genes, 2 ribosomal RNA (rRNA) genes, and 2 control regions (D-loop). The base composition was 36.7% for A, 12.5% for G, 27.2% for C, and 23.6% for T. The percentage of A + T (61.3%) reflected a typical sequence feature of the vertebrate mitogenome. Most of the *L. wui* genes are encoded on the H-strand except for the two *ND6* genes and eight tRNA genes, which are encoded on the L-strand. The positions of RNA genes were predicted by the MITOS (Bernt et al. 2013), and the locations of PCGs were identified by comparing with the homologous genes of other closely related species. Among the mitochondrial protein-coding genes, the *ATP8* was the shortest, while the *ND5* was the longest. The 12s rRNA is 881 bp long and the 16s rRNA is 1496 bp in length. The two rRNA are located between the *tRNA-Phe* and *tRNA-Leu* genes and separated by the *tRNA-Val* gene. The 22 tRNA genes range in size from 57 to 75 bp. The gene order, contents and base composition are identical to those found in typical vertebrates (Boore 1999; Sorenson et al. 1999).

Phylogenetic analysis was performed based on the complete mitogenomes of *L*. *wui* and together with other 13 related species from GenBank to further validate the newly determined sequences. These species were as follows: *Laudakia tuberculata*, *Phrynocephalus grumgrzimailoi*, *Phrynocephalus mystaceus*, *Phrynocephalus frontalis*, *Phrynocephalus przewalskii*, *Phrynocephalus albolineayuts*, *Xenagama taylori*, *Hydrosaurus amboinensis*, *Pogona vitticeps*, *Pseudotrapelus sinaitus*, *Chlamydosaurus kingii*, *Acanthosaura armata*, *Leiolepis belliana* (out group). We aligned these sequences using Clustal X (Thompson et al. 1997). A maximum-likelihood (ML) tree was constructed based on the dataset by the online tool RAxML (Stamatakis et al. 2008). The phylogenetic analysis result was consistent with the previous research with high support. It indicated that our new determined mitogenome sequences could meet the demands and explain some evolution issues.

**Figure 1.**
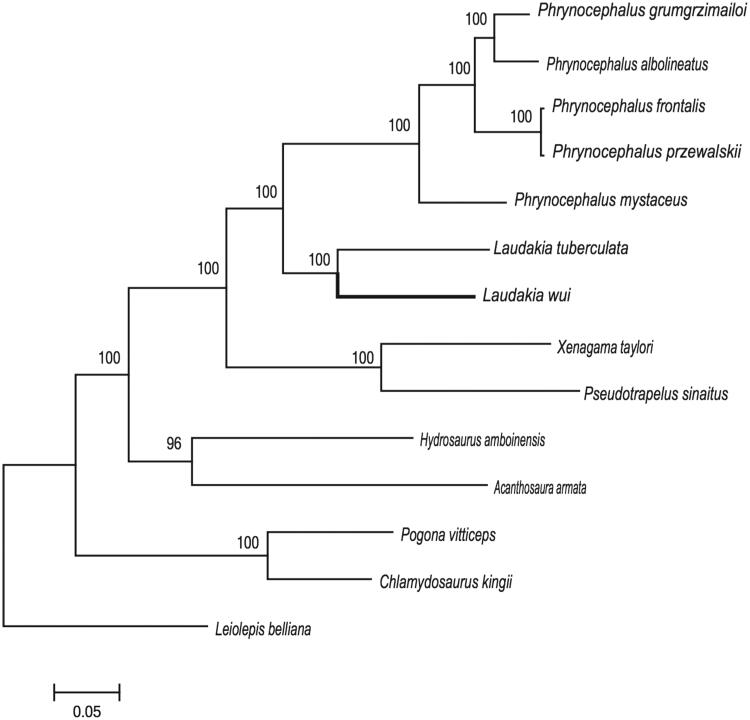
A maximum likelihood (ML) tree of *L. wui* in this study and other related species was constructed based on the dataset of the whole mitochondrial genome by online tool RAxML. The numbers above the branch meant bootstrap value. Bold black branches highlighted the study species and corresponding phylogenetic classification. The analyzed species and corresponding NCBI accession number as follows: *Laudakia tuberculata* (MK411595), *Phrynocephalus grumgrzimailoi* (KM093859), *Phrynocephalus mystaceus* (KC578685), *Phrynocephalus frontalis* (MF039064), *Phrynocephalus przewalskii* (MF039064), *Phrynocephalus albolineatus* (KP279760), *Xenagama taylori* (DQ008215), *Hydrosaurus amboinensis* (AB475096), *Pogona vitticeps* (AB475096), *Pseudotrapelus sinaitus* (AB262447), *Chlamydosaurus kingii* (EF090422), *Acanthosaura armata* (EF090422), *Leiolepis belliana* (AB537554).

## References

[CIT0001] Bernt M, Donath A, Juhling F, Externbrink F, Florentz C, Fritzsch G, Putz J, Middendorf M, Stadler PF 2013 MITOS: improved de novo metazoan mitochondrial genome annotation. Mol Phylogenet Evol. 69:313–319. 22982435 10.1016/j.ympev.2012.08.023

[CIT0002] Boore JL 1999 Animal mitochondrial genomes. Nucl Acids Res. 27:1767–1780. 10101183 10.1093/nar/27.8.1767 PMC148383

[CIT0003] Hu SQ, Zhao EM, Jiang YM, Fei L, Ye CY, Hu QX, Huang QY, Huang YZ, Tian WS 1987 Amphibia-Reptilia of Xizang. Beijing (China): Science Press; p. 107–109.

[CIT0004] Jiang ZG, Jiang JP, Wang YZ, Zhang E, Zhang YY, Li LL, Xie F, Cai B, Cao L, Zheng GM, et al. 2016 Red List of China’s vertebrates. Biodiversity Science. 24:500–951.

[CIT0005] Sorenson MD, Ast JC, Dimcheff DE, Yuri T, Mindell DP 1999 Primers for a PCR-based approach to mitochondrial genome sequencing in birds and other vertebrates. Mol Phylogenet Evol. 12:105–114. 10381314 10.1006/mpev.1998.0602

[CIT0006] Stamatakis A, Hoover P, Rougemont J 2008 A rapid bootstrap algorithm for the RAxML Web servers. Syst Biol. 57:758–771. 18853362 10.1080/10635150802429642

[CIT0007] Thompson JD, Gibson TJ, Plewniak F, Jeanmougin F, Higgins DG 1997 The CLUSTAL_X windows interface: flexible strategies for multiple sequence alignment aided by quality analysis tools. Nucleic Acids Res. 25:4876–4882. 9396791 10.1093/nar/25.24.4876 PMC147148

[CIT0008] Zhao EM 1998 A new species of the genus *Laudakia* from Xizang (Tibet) autonomous region. Acta Zootaxonomica Sinica. 23:440–444.

[CIT0009] Zhao EM, Zhao KT, Zhou KY 1999 Fauna Sinica, Reptilia. Beijing (China): Science Press; p. 133–147.

